# Identifying the neural network for neuromodulation in epilepsy through connectomics and graphs

**DOI:** 10.1093/braincomms/fcac092

**Published:** 2022-04-06

**Authors:** Artur Vetkas, Jürgen Germann, Gavin Elias, Aaron Loh, Alexandre Boutet, Kazuaki Yamamoto, Can Sarica, Nardin Samuel, Vanessa Milano, Anton Fomenko, Brendan Santyr, Jordy Tasserie, Dave Gwun, Hyun Ho Jung, Taufik Valiante, George M Ibrahim, Richard Wennberg, Suneil K Kalia, Andres M Lozano

**Affiliations:** 1 Division of Neurosurgery, Toronto Western Hospital, University Health Network, University of Toronto, 399 Bathurst St, Toronto, ON M5T 2S8, Canada; 2 Neurology Clinic, Department of Neurosurgery, Tartu University Hospital, University of Tartu, Tartu 50406, Estonia; 3 Joint Department of Medical Imaging, University of Toronto, Toronto, ON M5T1W7, Canada; 4 Section of Neurosurgery, Health Sciences Centre, University of Manitoba, Winnipeg, MB R3T2N2, Canada; 5 Schulich School of Medicine and Dentistry, Western University, London, ON N6A5C1, Canada; 6 Department of Neurosurgery, Yonsei University College of Medicine, Seoul 03722, Republic of Korea; 7 Krembil Research Institute, 399 Bathurst St, Toronto, ON M5T 2S8, Canada; 8 CRANIA, University Health Network and University of Toronto, Toronto, ON M5G 2A2, Canada; 9 The KITE Research Institute, University Health Network, Toronto, ON M5G 2A2, Canada; 10 Division of Pediatric Neurosurgery, Sick Kids Toronto, University of Toronto, Toronto, ON M5G1X8, Canada

**Keywords:** epilepsy, deep brain stimulation, network, connectome, biomarker

## Abstract

Deep brain stimulation is a treatment option for patients with drug-resistant epilepsy. The precise mechanism of neuromodulation in epilepsy is unknown, and biomarkers are needed for optimizing treatment. The aim of this study was to describe the neural network associated with deep brain stimulation targets for epilepsy and to explore its potential application as a novel biomarker for neuromodulation. Using seed-to-voxel functional connectivity maps, weighted by seizure outcomes, brain areas associated with stimulation were identified in normative resting state functional scans of 1000 individuals. To pinpoint specific regions in the normative epilepsy deep brain stimulation network, we examined overlapping areas of functional connectivity between the anterior thalamic nucleus, centromedian thalamic nucleus, hippocampus and less studied epilepsy deep brain stimulation targets. Graph network analysis was used to describe the relationship between regions in the identified network. Furthermore, we examined the associations of the epilepsy deep brain stimulation network with disease pathophysiology, canonical resting state networks and findings from a systematic review of resting state functional MRI studies in epilepsy deep brain stimulation patients. Cortical nodes identified in the normative epilepsy deep brain stimulation network were in the anterior and posterior cingulate, medial frontal and sensorimotor cortices, frontal operculum and bilateral insulae. Subcortical nodes of the network were in the basal ganglia, mesencephalon, basal forebrain and cerebellum. Anterior thalamic nucleus was identified as a central hub in the network with the highest betweenness and closeness values, while centromedian thalamic nucleus and hippocampus showed average centrality values. The caudate nucleus and mammillothalamic tract also displayed high centrality values. The anterior cingulate cortex was identified as an important cortical hub associated with the effect of deep brain stimulation in epilepsy. The neural network of deep brain stimulation targets shared hubs with known epileptic networks and brain regions involved in seizure propagation and generalization. Two cortical clusters identified in the epilepsy deep brain stimulation network included regions corresponding to resting state networks, mainly the default mode and salience networks. Our results were concordant with findings from a systematic review of resting state functional MRI studies in patients with deep brain stimulation for epilepsy. Our findings suggest that the various epilepsy deep brain stimulation targets share a common cortico-subcortical network, which might in part underpin the antiseizure effects of stimulation. Interindividual differences in this network functional connectivity could potentially be used as biomarkers in selection of patients, stimulation parameters and neuromodulation targets.

## Teaser

Vetkas et al. identified a brain network common to neuromodulation targets for epilepsy based on normative functional connectivity. The cortico-subcortical network might underpin the mechanisms of neuromodulation in epilepsy and pathophysiology. Connectivity between described hubs could be potentially used as a biomarker for selection of patients, neuromodulation targets and parameters.

## Graphical Abstract

**Graphical Abstract:**
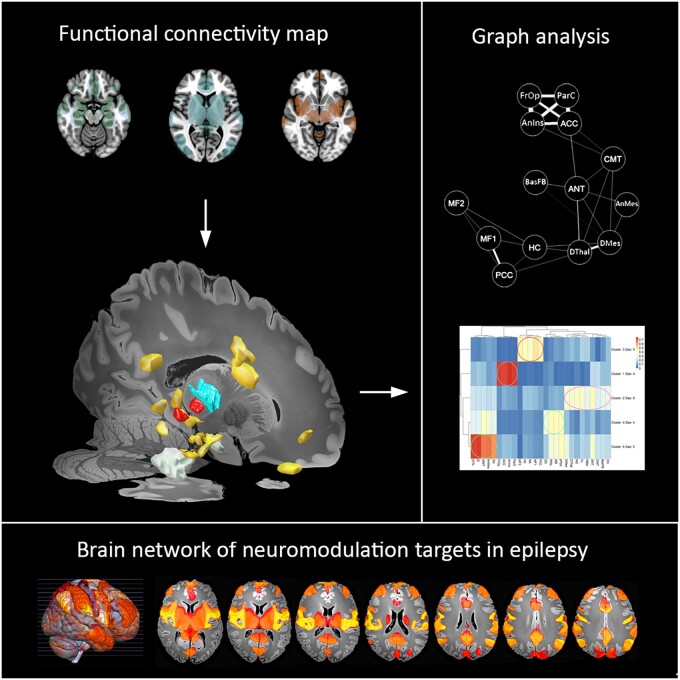

## Introduction

Deep brain stimulation (DBS) is an alternative or adjuvant treatment for patients with drug-resistant epilepsy (DRE) who are not candidates for resective surgery. Approximately 30% of patients with epilepsy continue to have debilitating seizures despite best medical managment.^1^ DBS has the potential to stop the propagation of epileptic seizures or increase the threshold for generalization.^2^ Possible mechanisms of DBS include activation of axons, local inhibition, effects on astrocytes, and disturbance of network oscillations.^2^

**Figure 1:**
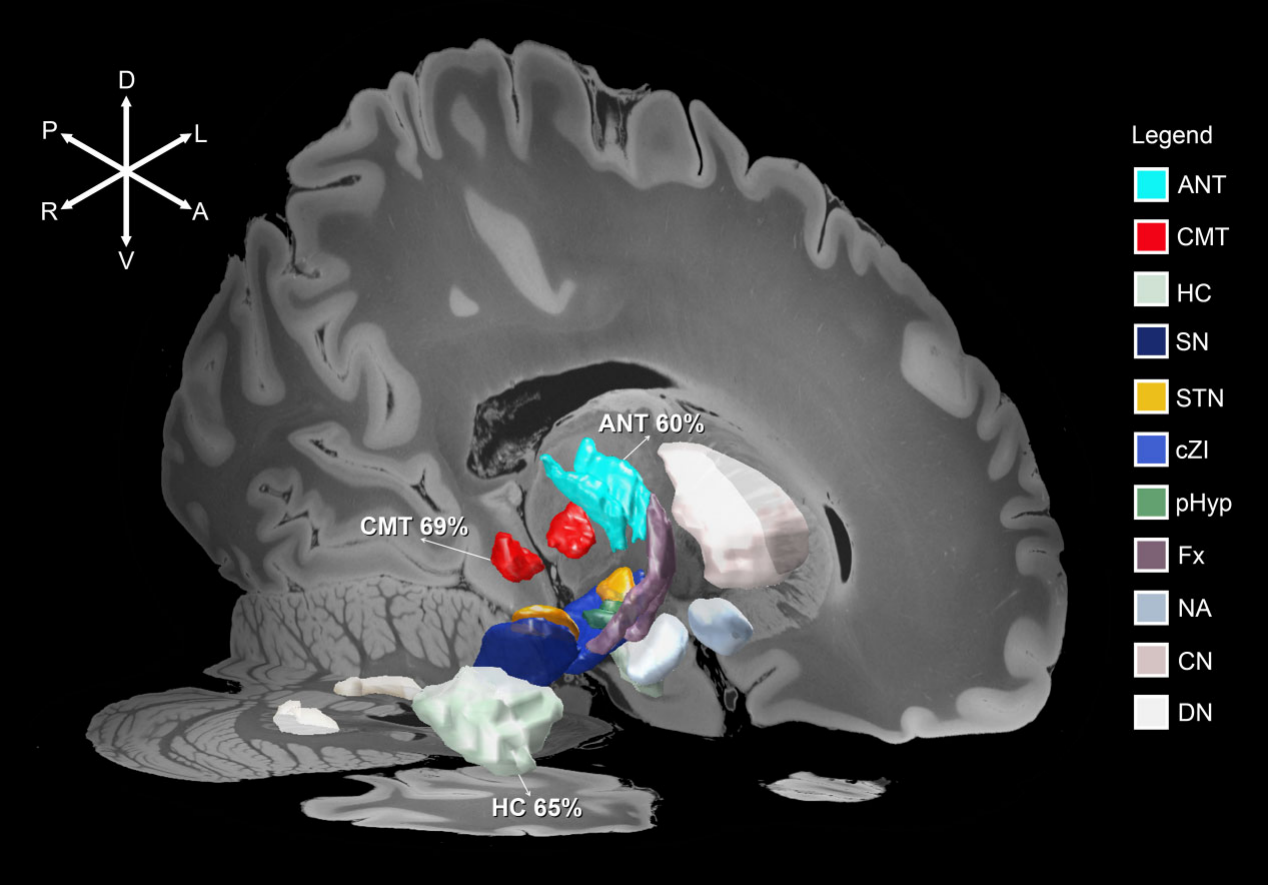
**Targets for DBS in epilepsy.** 3D representation of anatomical seeds of DBS targets used for the treatment of epilepsy is shown on sagittal and axial 7-Tesla T1 MRI slices of the human brain (100 μm resolution in MNI152 space). Mean seizure reduction that was calculated from our systematic literature review is shown for ANT – 60%, CMT – 69%, and HC – 65%. Based on our systematic review, higher quality of evidence exists for the use of ANT-DBS in DRE. Other DBS targets that belong to known cortico-subcortical circuits are visualized on the figure.

Various brain targets have been explored for DBS in epilepsy. Clinically used targets include the anterior nucleus of the thalamus (ANT), centromedian nucleus of the thalamus (CMT) and the hippocampus (HC), all of which have been investigated in randomized controlled trials.^3^ ANT-DBS received Food and Drug Administration approval in adult focal DRE. A randomized trial (SANTE) reported a median seizure reduction (SR) of 40% in patients who received active stimulation for 3 months, compared to 15% in the control group.^4^ In SANTE’s long-term follow-up, a median SR of 75% was reported at 7 years.^5^ CMT-DBS, in contrast, has been commonly used in patients with generalized epilepsies, particularly in the pediatric population.^6,7^ Hippocampal DBS has been performed in patients with seizures originating from the temporal lobe.^8,9^ Smaller, uncontrolled studies have probed the therapeutic potential of numerous other targets involving the circuit of Papez, limbic or cortico-subcortical circuits, including the subthalamic nucleus (STN), substantia nigra pars reticulata (SNr), caudal zona incerta (cZI), posterior hypothalamus (PH), fornix (Fx), nucleus accumbens (NAs), head of caudate nucleus (HCN), dentate nucleus (DN) and cerebellum (CB) (Fig. 1).^3^ Additional hypothetical targets for DBS in epilepsy have been studied in animal research.^3^

Disruption or modulation of epileptogenic networks most likely plays an important role in the therapeutic effect of DBS.^10–12^ Clinical efficacy has been associated with multiple DBS targets that belong to different anatomical circuits (motor, limbic, memory), suggesting the possibility of a common functional neural network responsible for the antiseizure effect associated with stimulation in epilepsy.^13^ Altered functional connectivity in patients with epilepsy, including deactivation of the default mode network (DMN), was described in simultaneous electroencephalography/functional MRI (EEG/fMRI) and MEG studies.^14–17^ Brain activity measured with blood oxygen level-dependent (BOLD) signal during focal epileptic discharges is significantly altered in wide cortical and subcortical regions that extend beyond the epileptogenic zone.^15^ Alterations in resting state functional connectivity, such as activation of DMN and limbic networks, are described in patients receiving DBS for epilepsy.^18–23^ Other treatment options for DRE such as vagal nerve stimulation (VNS), responsive neural stimulation (RNS) and resective surgery also lead to reorganization of neural networks.^24–26^ Data gathered from EEG/fMRI and MEG studies is used in the evaluation of epileptic patients and network analysis techniques can be applied to surgical planning.^25,27^ The importance of functional and structural connectivity has gained recognition in neurologic and psychiatric disorders such as Parkinson’s disease, Alzheimer’s disease, obsessive-compulsive disorder and epilepsy, and the term circuitopathies has been introduced to describe the effects of neural network alterations in disease and treatment.^28,29^ Epilepsy is considered to be a network disease with different cortico-subcortical hubs responsible for seizure evolution and maintenance.^14^

The aim of this study was to describe the functional brain networks associated with clinically important DBS targets for epilepsy (ANT, CMT, HC) and to examine how these networks relate: (i) to each other; (ii) to patterns of connectivity associated with less studied epilepsy DBS targets and with epilepsy pathophysiology; and (iii) to canonical resting state networks. We hypothesized that ANT, CMT and HC targets would exhibit connectomic overlap and that their functional networks would overlap with known epileptic circuits and with the DMN.

## Materials and methods

PubMed database screening was performed using the terms ‘seizure deep brain stimulation’ and ‘epilepsy deep brain stimulation’. Seizure outcomes for ANT, CMT and HC were gathered from clinical trials that included more than three patients and were published during the last two decades. Seizure outcomes for the less used targets were collected from all identified studies, and hypothetical DBS targets were screened during review. Studies included in calculation of means and identified targets are presented in Supplementary material 1. Mean SR was estimated from medians in studies where means were not reported.^30,31^ Response rate was determined as SR of 50% compared to baseline. DBS targets included in the analysis were classified as: clinically used epilepsy DBS targets (ANT, CMT, HC), less used DBS targets (STN, SNr, cZI, PH, Fx, NA, HCN, DN) and hypothetical targets [mammillothalamic tract (MMT), mammillary body (MB), nucleus basalis of Meynert, pedunculopontine nucleus (PPN) and medial septum]. Seeds of DBS targets were created from anatomical atlases (Supplementary material 1). A previously published ANT seed was used that covered the anterior nuclei targeted by DBS.^23^

### Statistical and connectivity analysis

Functional connectivity maps were created for each DBS target region to investigate the brain network associated with SR following DBS. Connectomic analysis has been previously used to describe neural networks associated with neuromodulation and neurological disorders.^32–34^ Functional connectivity maps for each seed (each DBS target) were calculated using a high-quality normative 3 T resting state fMRI data set derived from 1000 healthy subjects (age range: 18–35 years; 57.6% female) of the Brain Genomics Superstruct Project (BGSP, https://dataverse.harvard.edu/dataverse/GSP), as previously described.^35–39^ The MRI acquisition and pre-processing parameters for the BGSP normative connectome are described in detail in the original publication.^40^ In short, the data were collected on 3 T Tim Trio scanners (Siemens, Erlangen, Germany) using a 12-channel phased-array head coil. The following EPI parameters were used: repetition time = 3000 ms; echo time = 30 ms; flip angle = 85°; voxel size = 3 × 3 × 3 mm; field of view = 216; slice acquisition = 47 axial slices acquired in interleaved fashion with no gap between slices. Each participant in the GSP study underwent one or two functional runs (mean of 1.7 runs), each of which lasted 6.2 min (124 time points). The fMRI preprocessing consisted of common methods: the first four volumes of each run were discarded, slice acquisition-dependent time shifts per volume were compensated for, and motion correction was applied. Temporal filtering was also performed, retaining frequencies below 0.08 Hz, and individual scans were normalized to common space. Finally, spatial smoothing of the resting-state data was performed.

Using the DBS target seeds whole-brain connectivity r-maps were generated describing the average correlation of the low-frequency BOLD signal fluctuation between the seed and each voxel. The voxel-wise r-maps show the average voxel to seed correlation across the entire normative dataset. To identify all voxels that meaningfully connected to each seed, the r-maps were converted to t-maps and using the known p-distribution these t-maps were Bonferroni corrected for multiple comparisons at |t| = 5.1 (Bonferroni corrected < 0.05, whole brain). The thresholded t-maps were binarized to capture the brain-wide pattern of regions significantly connected with each seed. Using this conservative approach subsequent results are not driven by subtle variations in local connectivity.^38^

**Figure 2:**
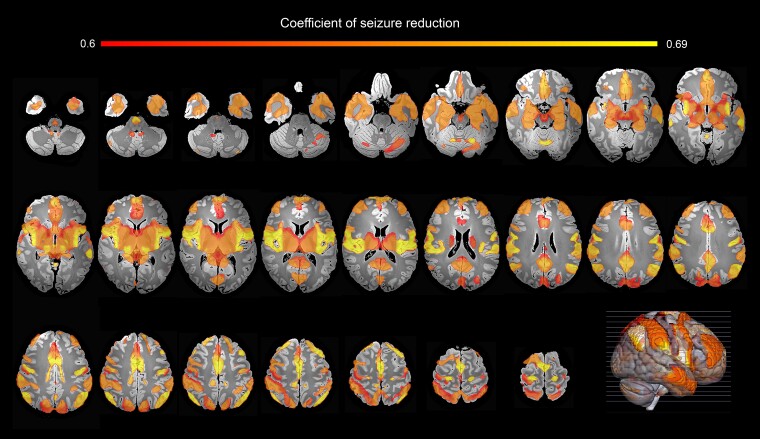
**Functional connectivity of clinically used epilepsy DBS targets (ANT, CMT, HC).** To identify the brain regions associated with DBS and symptom improvement, a weight equal to targets mean seizure reduction was assigned to the binarized connectivity t-maps calculated from normative resting state fMRI scans of 1000 individuals, and a voxel efficacy map of percent improvement of ANT, CMT, and HC was created. Areas of functional connectivity affected by DBS are shown on the axial 7-Tesla T1 MRI slices. *A* 3D figure with an overlay of the functional connectivity map represents the level of the axial slices. Cortical regions affected by DBS for epilepsy are the ACC, PCC, insula, medial and lateral frontal region, temporal lobe, superior parietal lobe, angular and supramarginal gyrus, cuneus, sensorimotor and premotor cortex. Subcortical regions affected by stimulation are the basal ganglia, dorsal and ventral mesencephalon, pons and CB.

To identify the brain regions associated with DBS and symptom improvement, a weight of mean SR was assigned to the functional maps, and a voxel efficacy map of percent improvement of ANT, CMT, and HC functional connectivity was created (Fig. 2; in-house MATLAB script, version R2017b; MathWorks, Natick, MA).^41^

**Figure 3:**
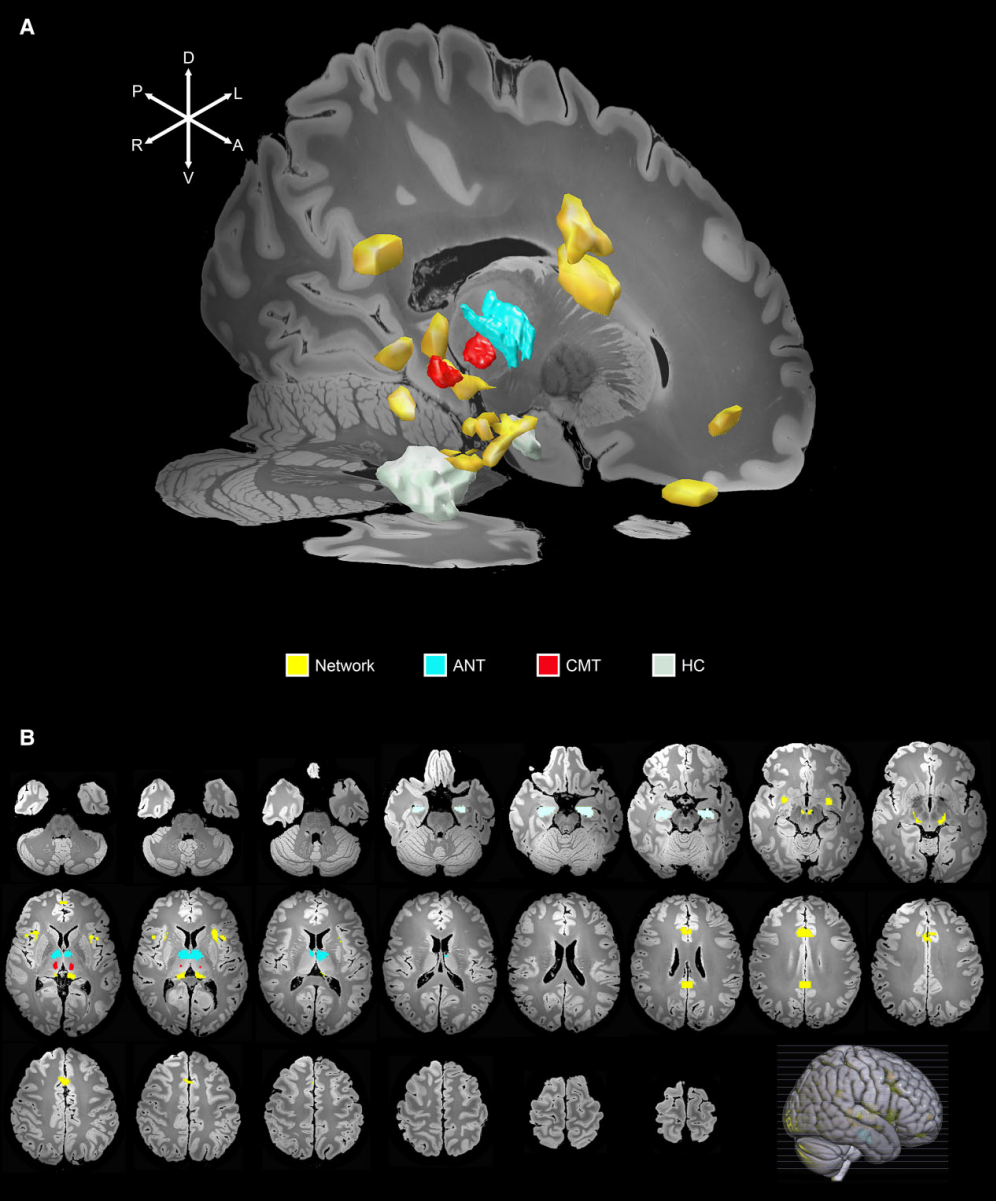
**Common brain network of deep brain stimulation in epilepsy.** (**A**) A 3D representation of the anatomical seeds of the clinically used targets for DBS in epilepsy (ANT, CMT, HC) and regions of their functional connectivity overlap calculated from resting state fMRI scans of 1000 individuals are shown on high-resolution sagittal and axial 7-tesla T1 MRI slices of the human brain. The regions that overlapped between the three functional connectivity maps of ANT, CMT, and HC (in yellow) were used to outline the common neural network implicated in seizure reduction following DBS. (**B**) The regions identified as the normative network model associated with common epilepsy DBS targets are shown on axial 7-Tesla T1 MRI slices. A 3-D figure with an overlay of the network represents the level of the axial slices. Regions of overlap were in the ACC, paracingulate cortex, medial frontal region, PCC, anterior insula, frontal operculum, right sensorimotor cortex, basal forebrain, medial thalamus, dorsal thalamus, dorsal and ventral mesencephalon.

The overlapping regions between the three binarized maps (ANT, CMT and HC) were used to outline the common network implicated in SR following DBS (the PCC and the frontobasal seed were averaged in size with other seeds for comparison). The identified regions were used to construct a network associated with DBS in epilepsy (Supplementary Fig. 1). Additional binarized maps of regions of high overlap were calculated for the subsequent network analysis (Fig. 3). For all seeds and high overlap regions, a Pearson correlation matrix was created between regions of overlapping functional connectivity and targets for DBS in epilepsy using Lead Mapper v2.5.3.^42^ Clustering of the Pearson correlation matrix of functional connectivity between brain regions included in the network was performed with the R package pheatmap (version 1.0.12) based on Euclidian distances where 5 clusters were obvious and chosen for illustration (the simple input correlation matrix is presented as Supplementary Fig. 2). The weighted network graph was created based on the functional connectivity correlation matrix with qGraph library (version 3.1.1) using the ‘qgraph’ function in R (https://www.r-project.org/, version 4.1.1). The regions of anticorrelation were not included in the graph analysis as per Rubinov and Sporns.^43^ Edge thickness denotes the strength of correlations between nodes (edge thickness cut-off was set at 0.3). Graph centrality measures (betweenness, closeness) were calculated. Higher betweenness shows how often a node is part of the shortest connection between regions and indicates a stronger influence on the transfer of information within the neural network. Closeness describes the average shortest distance from each node in the network to others and reflects how efficiently a node can spread information within a network.^44^

To compare the normative network model associated with common epilepsy DBS targets with reports from patient studies, we performed a systematic review of resting state fMRI findings in epilepsy DBS (three patient reports and three normative fMRI studies). PubMed search terms ‘epilepsy DBS functional MRI’, ‘epilepsy DBS fMRI’, ‘epilepsy deep brain stimulation fMRI’, were used, and bibliographies of identified articles were searched for additional reports.

### Data availability

All data is available upon reasonable request.

## Results

### Seizure outcomes in DBS for epilepsy

The calculated mean SR after DBS of ANT, CMT and HC in clinical trials involving more than three patients was 60, 69 and 65%, respectively. DBS of ANT is used more often for focal epilepsies of temporal and extratemporal origin, CMT for generalized epilepsies and HC for temporal lobe epilepsy. Higher quality evidence exists for DBS of ANT than for CMT or HC.^45^ Outcomes for less studied epilepsy DBS targets vary and range from 48 to 92% (Table 1). Initial studies of cerebellar DBS showed promising results, but a subsequent randomized trial did not achieve statistical significance.^13^

**Table 1:** Review of outcomes for DBS in epilepsy. Seizure reduction, response rate (SR > 50%), and cohort characteristics of patients with deep brain stimulation for epilepsy were extracted from a systematic literature review

Target	*N*	Mean age	Follow-up (mo)	Mean seizure reduction	Number of studies
ANT	330	33.5	34.2	59.6	23
CMT	90	23.9	28.7	69.3	8
HC	107	33.8	33.9	64.6	13
STN	22	21.7	26.2	66.5	9
cZI	6	34.7	38.2	87.5 SR in two studies, 3/3 RR in one study	3
SN	1	32	24	100 in GTC, improvement in myoclonic seizures	1
PH	7	24.5	47.3	83.6	2
Fx	7	41	1-9 days	92	1
NA	9	39.5	6	47.5	2
CN	38	None	18	21/38 SF, 14/38 improved	1
DN	95	28.6	36.3	Mixed results	8

### Areas of functional connectivity associated with DBS in epilepsy

A summed map of ANT, CMT and HC functional connectivity weighted by mean seizure outcome revealed wide cortical and subcortical regions affected by stimulation. Cortical areas with higher time course correlation to DBS targets were in the anterior cingulate cortex (ACC), posterior cingulate cortex (PCC), insula, medial and lateral frontal region, temporal lobe, superior parietal lobe, angular and supramarginal gyrus, cuneus, sensorimotor and premotor cortex. Subcortical areas with higher time course correlation were in the basal ganglia, dorsal and ventral mesencephalon, pons and CB (Fig. 2).

### Common brain network of DBS in epilepsy

The overlap of the maps of voxels that are significantly connected to the epilepsy DBS targets used for successful treatment overlaps highly and outlines a common brain network underlying treatment (Supplementary Fig. 1). Functional connectivity maps of ANT, CMT and HC seeds had regions of overlap in the ACC, paracingulate cortex, medial frontal region, PCC, anterior insula, frontal operculum, right sensorimotor cortex, basal forebrain, medial thalamus, dorsal thalamus, dorsal and ventral mesencephalon. DBS targets and areas of overlapping functional connectivity between them were identified as a common neural network associated with the action of DBS in epilepsy (Fig. 3). Cortical and subcortical regions of overlapping functional connectivity between clinically used, less used, and hypothetical DBS targets were compared and are presented in Supplementary Table 1.

### Graph analysis of the epilepsy DBS network

**Figure 4:**
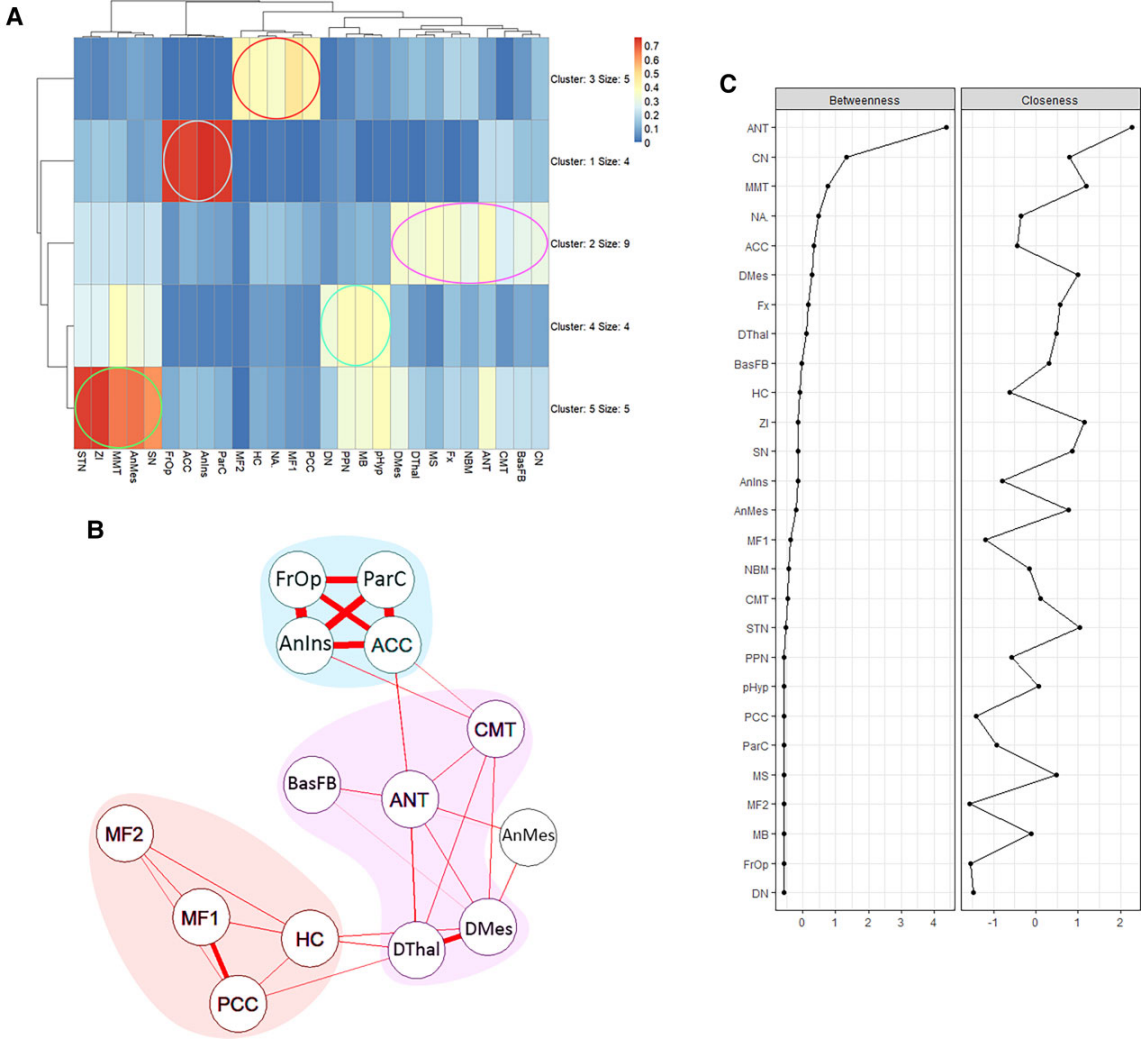
**Graph analysis of the normative epilepsy DBS network.** (**A**) A Pearson correlation matrix of functional connectivity between seeds (ANT, CMT, HC, areas of common functional connectivity, less studied and hypothetical DBS targets) was clustered into 5 groups based on the strength of correlations to present the associations between regions involved in DBS for epilepsy. (**B**) A graph network, based on the correlation matrix between the epilepsy DBS targets (ANT, CMT, HC) and regions of their functional connectivity overlap, was created to visualize the relationship between nodes in the epilepsy DBS neural network. Thickness of edges shows the strength of correlations between the nodes (minimum correlations presented >0.2). **C**. Centrality measures of the epilepsy DBS neural network show that ANT, CN, and MMT act as a central hubs in the network with the highest betweenness and closeness values, while CMT and HC showed average centrality values (standardised z-scores on the x-axis).

A correlation matrix of functional connectivity, based on normative resting state fMRI, showed clustering of nodes into five groups (Fig. 4A). Graph analysis revealed a network with two cortical clusters and a subcortical cluster (Fig. 4B). The first cluster largely corresponded to cortical regions of the DMN (medial frontal cortex, PCC, HC). This group of nodes had stronger correlations to dorsal thalamus and dorsal mesencephalon. The second cluster included cortical regions involved in the saliency network (ACC and paracingulate cortex, frontal operculum, and anterior insula) and had stronger correlations with ANT and CMT. The largest cluster included ANT, CMT, most other DBS targets and subcortical nuclei. A separate cluster of subcortical regions included the anterior mesencephalon structures and the MMT. The fifth cluster contained PPN, PH, MB and DN. The anterior thalamic nucleus had a central position in the network with the highest betweenness and closeness values showing more connections to other nodes in network, while CMT and HC had average centrality values (Fig. 4C). Caudate nucleus and MMT also displayed high centrality values. ACC displayed the highest centrality values from the cortical regions. The expanded graph network with the less used and hypothetical DBS targets is presented in Supplementary Fig. 3.

### Review of resting state fMRI studies in patients with DBS for epilepsy

Several recent studies examined the resting state fMRI changes in patients with DBS and showed associations with functional connectivity in the DMN, sensorimotor cortex and other regions (Table 2). A study using resting state fMRI in two ANT DBS patients with DBS on showed activation in regions associated with the limbic and DMNs of the brain.^21^ Functional connectivity associated with regions of DMN was reported from electrode volume of tissue activated (VTA) analyses in three ANT DBS responders.^20^ The association of ANT stimulation with DMN was further confirmed in patients with long-term ANT DBS.^23^ A recent study with low- and high-frequency stimulation during resting state fMRI showed that high frequency DBS produced activation of DMN and limbic networks of the brain, in contrast to low-frequency stimulation.^22^ Studies of functional connectivity associated with CMT electrode VTAs produced less functional connectivity in regions of DMN, but higher connectivity with sensorimotor, premotor, ACC, ascending reticular activating system (ARAS) and other brain regions (Table 2).^18, 19^

**Table 2:** Review of patient and normative (VTA based) resting state fMRI studies in DBS for epilepsy

Study	Target (*N*)	fMRI details	Main finding
Middlebrooks *et al.* 2018^20^	ANT (6)	Electrode VTAs, normative	Responders had connectivity in regions of posterior cingulate cortex, medial prefrontal cortex, inferior parietal lobule and precuneus, which are associated with DMN.
Middlebrooks et al. 2020^21^	ANT (2)	ON/OFF in 30 s blocks	Activation in bilateral thalamus, anterior cingulate and posterior cingulate cortex, precuneus, medial prefrontal cortex, amygdala, ventral tegmental area, HC, striatum and right angular gyrus
Middlebrooks *et al.* 2021^22^	ANT (5)	ON/OFF in 30 s blocks	High-frequency stimulation led to activation in DMN (PCC, precuneus, angular gyrus, parahippocampal gyrus, medial thalamus, and basal forebrain) and limbic (HC, ACC, amygdala, ANT, medial prefrontal cortex, and ventral tegmental area areas). Low-frequency stimulation produced deactivation in the similar regions.
Sarica *et al.* 2020^23^	ANT (2)	ON/OFF in 30 s blocks	Stimulation of ANT contacts produced positive connectivity with bilateral putamen, thalamus, and posterior cingulate cortex, ipsilateral middle cingulate cortex and precuneus, and contralateral medial prefrontal and anterior cingulate cortex
Warren *et al.* 2020^19^	CMT (19)	Electrode VTAs, normative	Postitive subcortical connectivity in cerebellum, thalamus, brainstem, striatum and subthalamic nuclei. Positive cortical connectivity in auditory cortex, precentral and postcentral gyri, premotor cortex, cingulate cortex, parahippocampal/fusiform cortex and insular cortex.
Diaz *et al.* 2021^18^	CMT (10)	Electrode VTAs, normative	Positive subcortical connectivity in thalamus, striatum and STN. Positive cortical connectivity in sensorimotor cortex, SMA, middle frontal cortex, medial temporal cortex, and anterior cingulate. Reponders had DTI fibres passing through VTAs connected to sensorimotor, supplementary motor cortex, middle and superior frontal gyrus, cerebellum and ARAS.

## Discussion

We used the seeds of epilepsy DBS targets and areas of their common functional connectivity, as defined from normative resting state fMRI scans of 1000 individuals, to identify a network associated with neuromodulation in epilepsy. Our results showed that epilepsy DBS targets have common areas of cortical and subcortical resting state functional connectivity in regions associated with propagation and generalization of epileptic seizures. The normative neural network shared by ANT, CMT, HC and other DBS targets overlapped cortical and subcortical regions associated with resting state networks, mainly the DMN and salience networks. The common functional connectivity network shared by clinically used DBS targets overlapped regions of the epileptic networks identified by previous studies of regions activated during DBS.^14,15,22,23^ This suggests that DBS targets and epilepsy share a common cortico-subcortical network that might be responsible for the antiseizure action of DBS.^21,46,47^

### Identifying the neural network associated with DBS in epilepsy

Cortical nodes identified in both the normative network model and associated with common epilepsy DBS targets were in the ACC and PCC, medial frontal and sensorimotor cortex, frontal operculum and bilateral insulae. Subcortical nodes of the network were in the basal ganglia, mesencephalon, basal forebrain and CB.

Clustering of the functional connectivity matrix showed two cortical groups and intercorrelated subcortical clusters that included most DBS targets. The first cortical group corresponded to nodes associated with the DMN (medial frontal cortex, PCC and precuneus, HC).^48^ The second cortical group consisted of nodes associated with the saliency network (ACC, anterior insula, frontal operculum).^49^ Overlap between ANT, CMT, and HC functional connectivity in the sensorimotor cortex occurred only on the right side. The sensorimotor cortex had a higher time course correlation with CMT and is associated with response to DBS in generalized epilepsy.^18^ Areas of resting state functional connectivity activated during successful DBS for epilepsy in patients reviewed from published studies (Table 2 of results) are similar to the network described in our study, suggesting that successful DBS leads to modulation of this network.

We found subcortical areas of common functional connectivity between ANT, CMT and HC in the medial and dorsal thalamus, dorsal and ventral mesencephalon, and CB, regions that have been previously implicated in the pathophysiology of seizures.^5,50^ The region in the dorsal thalamus corresponded to medial pulvinar, and is associated with temporal lobe seizures and status epilepticus.^51–53^ Additionally, the pulvinar nucleus was recently successfully used as a target for responsive neural stimulation (closed loop technique) in patients with posterior quadrant epilepsy.^54^ The identified areas of functional connectivity in the basal forebrain, medial and mediodorsal thalamus are parts of the DMN, and are likely parts of a common subcortical circuit associated with DBS in epilepsy.^55^ The area in the superior dorsal mesencephalon (lateral geniculate body) is associated with epileptic seizures and antiseizure effects.^56–58^ Hyperactivity in the tegmentum is associated with loss of consciousness during seizures and following brain trauma.^47,59,60^ The role of pontine and mesencephalic ascending reticular formation in consciousness supports the cortico-subcortical framework of seizure generalization in epilepsy.^2^ A recent study of CMT DBS showed an association with better outcome when ARAS was covered by the VTAs of electrodes.^18^ The long-term follow-up of ANT stimulation in the SANTE trial showed a decrease in sudden unexpected death in epilepsy (SUDEP), which suggests the possibility that this might be related to the effect of stimulation on the brainstem Raphe nuclei.^5^

### Graph analysis of the epilepsy DBS network

Graph analysis revealed ANT as a central hub in the normative network model associated with common epilepsy DBS targets with the highest betweenness and closeness centrality values. Mammillothalamic tract shared similar centrality values to ANT. Simultaneous targeting of MMT and ANT is associated with better outcomes after DBS in epilepsy.^61^ Adding MMT to the ANT seed increased the area of functional correlation in the brainstem and ACC region.

The anterior thalamic nucleus and CMT had a stronger correlation with ACC, dorsal thalamus, and mesencephalon, which are likely important hubs in the neural network associated with DBS action in epilepsy. CMT receives input from ARAS, which may explain the effectiveness in generalized seizures associated with loss of consciousness. Other DBS targets with high centrality values are the caudate nucleus and NAs. The NAs had a bigger area of functional connectivity in the frontal lobes and had higher correlations with the frontal cortical cluster. The basal forebrain seed had a strong correlation with ANT, dorsal thalamus and dorsal mesencephalon, which potentially makes it a novel target for DBS for epilepsy. The DN had the lowest correlation to the network, which is reflected in its questionable clinical efficacy in DBS for epilepsy.

The ACC was identified as an important hub in the neural network associated with the effect of DBS in epilepsy. Low-frequency DBS (5 Hz) of CMT in 10 patients resulted in a sequential propagation of the EEG source from the ACC to frontal, then to temporal and other cortical regions.^62^ Similar activation in ACC was demonstrated by distributed source modelling of EEG recorded during DBS of ANT in three patients, and during stimulation of different anterior thalamic subnuclei in five patients.^63,64^ In our graph analysis ACC had a strong correlation with three cortical seeds (frontal operculum, paracingulate cortex, and anterior insula). In the identified normative network model associated with common epilepsy DBS targets ACC acted as a relay for DBS targets to the cortical cluster, similarly to the propagation of electrical signals in neurophysiological studies. Furthermore, enhanced intrinsic functional connectivity between the thalami, ACC and insula predicts response to VNS in children with DRE.^65^

### Association of the epilepsy DBS network with resting state fMRI networks

We described an overlap of normative functional connectivity between ANT, CMT and HC seeds in regions corresponding to the DMN. The DMN includes the the medial prefrontal cortex and ACC, PCC, precuneus, medial and lateral parietal cortices, and temporal areas, and is involved in introspection and social functions.^66^ A recent study identified additional regions associated with the DMN in the basal forebrain, and anterior and mediodorsal thalamus.^55^ The DMN plays an important role in generalized and focal epilepsy. Reduced functional connectivity between regions of DMN is reported in patients with idiopathic generalized epilepsy.^67^ Decreased fMRI activity in the DMN occurs in patients during bursts of generalized spike-and-wave (GSW) discharges in patients with primary generalized epilepsy.^68^ Deactivation of DMN identified by fMRI and single photon emission computerized tomography (SPECT) is associated with loss of consciousness during generalized and focal impaired awarness seizures (FIAS), which is consistent with the ‘network inhibition hypothesis’.^68,69^ Danielson *et al.*^70^ proposed that increased thalamic activity and inhibition of the reticulate activating system, which otherwise maintains the DMN, could be the common mechanism for loss of consiousness in seizures of different types (FIAS, generalized tonic-clonic, absence). Decrease in DMN connectivity also occurs in other pathological states of decreased consciousness, eg. in minimally conscious patients.^71,72^ Similarly, in a study of focal interictal epileptiform discharges in patients with temporal, frontal and posterior quadrant epilepsy, the common finding was deactivation in the DMN in all three groups, despite the large differences in localization of the epileptiform discharges.^73^ Patients with focal seizures exhibit fMRI changes not only in the seizure onset zone, but have wider alterations in functional connectivity of the brain during epileptiform discharges, including cortical and subcortical sites in thalamus, basal ganglia, and reticular activating system.^74^

The second cortical cluster in our graph analysis potentially corresponds to the cortical regions of the salience network (ACC, frontoinsular cortex and subcortical structures).^75^ Altered intrinsic connectivity in the salience network was described in patients with childhood absence epilepsy.^49^ Integrity of the salience network is associated with DMN function.^76,77^ ACC, medial frontal cortex, bilateral insulae, sensorimotor cortex and caudate nucleus have been implicated in initiation and propagation of ictal discharges together with the mediodorsal nuclei of thalamus in an EEG-fMRI study.^78^ The ACC has been functionally connected to the basal forebrain and DMN in recent human and animal studies.^55^ The ACC is an important part of the salience network. As part of the limbic and Papez circuits, ACC is associated with affection, cognition and seizure propagation.^79,80^ Decreased connectivity between thalamus and ACC, measured with positron emission tomography (PET), is associated with the minimally conscious state, and connectivity between them increases with clinical improvement.^81^ Children with epilepsy have better neurocognitive outcomes when resting state networks are not perturbed by interictal epileptiform discharges. Consequently, normalization of intrinsic network connectivity through DBS could lead to better cognitive and neuropsychiatric outcomes in patients with epilepsy.^82^

### Role of the anterior and centromedian thalamic nucleus, and HC in DBS

The anterior thalamic nucleus is the most commonly studied target for DBS in epilepsy patients with focal and secondarily generalized seizures.^5^ The anterior thalamic nucleus is part of the Papez circuit, a loop that consists of HC, Fx, mammillary nuclei, ANT and cingulum. The Papez circuit is involved in memory and cognition.^83^ Remote regions, such as CB, STN and CMT project to nodes within the circuit of Papez.^84^ The anterior thalamic nucleus has wide reciprocal connections to cingulum and the hippocampal area.^85^ The anterior thalamic nucleus is reported to be involved in maintenance and propagation of epileptic seizures.^86^ Multiple studies have reported an association between ANT stimulation and DMN alterations.^1,20,23^

The centromedian thalamic nucleus is involved in seizure propagation and is probably implicated in loss of consciousness during generalized seizures due to its anatomical connections to the ARAS and wide projections to the cortex, mainly premotor and motor regions.^86–88^ Higher reduction in generalized seizures after CMT-DBS was associated with stimulation affecting discriminative structural connectivity fibres overlapping ARAS, sensorimotor and supplementary motor cortices and CB/brainstem.^18^ The centromedian thalamic nucleus is activated earlier during generalized seizures than the ANT, suggesting that early seizure propagation might occur in posterior regions of the thalamus and subsequent maintenance of ictal activity in the anterior regions.^89^ The centromedian thalamic nucleus is likely also involved in focal seizures and their generalization, but studies reported a lower decrease in focal seizures after CMT stimulation.^6,90^ The centromedian thalamic nucleus stimulation was more effective for generalized than frontal lobe epilepsy in a study of 11 patients (84% mean SR in generalized seizures versus 47% mean SR in frontal seizures), which could be related to higher anatomical connectivity of CMT to premotor, motor, and primary somatosensory areas.^7,88^ Patients with genetic (primary) generalized epilepsy studied with EEG-fMRI during GSW discharges had a substantial BOLD activation in the striatum and motor/premotor cortex.^91^ In a recent study, functional connectivity associated with CMT-DBS electrode regions of interest showed an overlap with the functional network described for generalized epilepsy.^19^

In cases of temporal lobe epilepsy, HC stimulation could potentially stop seizure propagation by interrupting the early spread of ictal activity from the seizure onset zone to other regions associated with seizure maintenance (i.e. ANT).^92^

The association between basal ganglia, ARAS and resting state networks (DMN, salience network) in epileptic seizures provides a common physiological substrate for the antiseizure effect of neuromodulation in epilepsy.^18–21^ The nodes in the normative network model associated with common epilepsy DBS targets can have different functions, i.e. some nodes can be directly associated with seizure emergence and propagation, while others and white matter tracts can be relays to functional hubs in the neural circuit. The effect of DBS might be produced by local changes in key hubs leading to neural network modulation and plasticity. In the future, surgical targets and stimulation parameters in epilepsy DBS could be individually selected according to the connectivity of the hubs to patient specific epileptic networks. Alternatively, hubs in the identified normative network model associated with common epilepsy DBS targets could be used as targets for DBS, non-invasive neuromodulation, or for sensing electrodes in closed loop stimulation or even for the prediction of SUDEP.^93^

### Approach to epilepsy as a network disorder

Neural network alterations that occur in patients with epilepsy are described both in the ictal and interictal state.^94,95^ Ponten *et al.*^96^ have demonstrated that epileptic seizures are characterized by a loss of normal balance between local and global connectivity compared to the interictal state based on the correlations between EEG signals from different brain regions. A meta-analysis of functional and structural connectivity in epilepsy summarized that a pattern of increased local connectivity and decreased global connectivity is present in epilepsy.^97^ Hyper-connected and hypersynchronized brain regions, that are associated with the epileptogenic zone, are likely important hubs in the epilepsy networks, and resection of these regions is connected to seizure freedom.^98–100^ In addition, an altered level of activity and connectivity is present in physiological hubs, most commonly in the DMN.^94^ Both surgical and medical treatment modalities can lead to reorganization of neural connectivity in epilepsy.^24,26,101^ The current understanding of the mechanism of neuromodulation, such as DBS, supports the notion that functional treatments exert their effects through modulation of neural networks.^28^ The technique of neural network interrogation using connectivity of neuromodulation targets through normative datasets gives valuable insights into the neuroscientific basis of disease and treatment mechanisms.^102^ Previous research in Parkinson's disease showed that normative functional connectivity studies and patient specific fMRI connectivity have a good overlap.^103^ Furthermore, previous work has demonstrated that while age or disease processes are associated with detectable differences in connectivity between hub regions within networks, these do not change the general network structure.^104–106^ Furthermore, no general change in functional network structure is associated with treatment resistant epilepsy, according to a recent review.^95^ The general pyshiological hubs and structure of the networks are consistent between individuals, but topological changes are possible in the epileptic networks.^99^ As the core question of the present study was the identification of the brain network associated with epilepsy treatment, the use of normative data should not be detrimental to achieving this goal. The significance of the network hubs identified in the epilepsy DBS network can be explained by their role in epilepsy networks, and in seizure spread and maintenance, but it has to be further confirmed by examining the connectivity alterations in patients with epilepsy. The demonstration of the role functional and structural networks in neuromodulation for epilepsy requires extensive research in long-term prospective patient cohorts to establish its clinical significance.

### Limitations

The use of normative fMRI data has some limitations. First normative data does not include any information about the individual patients included in the specific study. Furthermore, since the normative dataset was acquired using healthy control subjects, any disease specific structural and functional abnormalities are also not reflected in the data. The notion of network reorganization in epilepsy (i.e. due to disease related pathological changes or antiseizure medication) cannot be accounted for in the current study. However, this approach allows for a solution to the complex issue of acquiring fMRI scans in a small DBS population with epilepsy and the typically encountered questionable test-retest reliability and poor signal to noise ratio in fMRI patient scans. While normative datasets cannot provide the same level of specificity as patients studies, previous research has demonstrated that analysis using either normative, patient-specific data or a disease specific connectome lead to the identification of the same brain networks.^39,103^ We described the epilepsy DBS network in normative scans, but our results are supported by fMRI-EEG studies in epilepsy patients, and by fMRI studies in patients with epilepsy and DBS. Using anatomical labels might lead to different results compared to active stimulation or VTAs of electrodes but allows for a standardized approach. Anatomical labels were selected to reduce the heterogenous results created by varying electrode positions. Combining targets that belong to different anatomical circuits (motor, limbic, Papez) does not allow identification of direct anatomical connections, but this approach allows for a study of shared functional connectivity that is present in the central nervous system based on the hodological framework.^107^ Our approach allowed us to describe the common functional connectivity network between DBS targets and epilepsy, with the limitation that we cannot pinpoint specific function of the nodes and their role in the therapeutic effect. Retrospective review of clinical studies can lead to bias (heterogenous groups of patients, differences in follow-up and programming, and stimulation parameters) and overestimation of clinical outcomes for less studied targets, an unavoidable limitation. Finally, the significance of our findings in different types of epilepsy, and for different subtypes of seizures requires further research in patient populations.

## Conclusion

We described a novel brain network associated with targets used for neuromodulation in epilepsy. The epilepsy DBS brain network shared hubs with known epileptic networks and regions involved in seizure propagation and generalization. The normative network model associated with common epilepsy DBS targets demonstrated a partial overlap with regions of the DMN and saliency network. Our results are supported by previous findings from fMRI studies in patients with DBS, RNS and VNS for epilepsy. Functional connectivity may be used as a biomarker in selection of targets or adjustment of DBS programming parameters. In the future, DBS treatment could be tailored to individual patients and disease-specific networks in epilepsy or other pathologies.

## Ethical publication statement

We confirm that we have read the Journal’s position on issues involved in ethical publication and affirm that this report is consistent with those guidelines.

## Supplementary Material

fcac092_Supplementary_Data

## Abbreviations

3D =: three-dimensional
ACC =: anterior cingulate cortex
ANT =: anterior nucleus of the thalamus
ARAS =: ascending reticular activating system
BOLD =: blood oxygenation level dependent imaging
cZI =: caudal zona incerta
CMT =: centromedian nucleus of the thalamus
DBS =: deep brain stimulation
DMN =: default mode network
DN =: dentate nucleus
CB =: cerebellum
Fx =: fornix
fMRI =: functional magnetic resonance imaging (fMRI)
HCN =: head of caudate nucleus
MEG =: magnetoencephalography
MB =: mammillary body
MMT =: mammillothalamic tract
MS =: medial septum (medial septum)
NA =: nucleus accumbens
NBM =: nucleus basalis of Meynert
PPN =: pedunculopontine nucleus
PCC =: posterior cingulate cortex
PH =: posterior hypothalamus
RR =: response rate
SF =: seizure free
SR =: seizure reduction
SPECT =: single photon emission computed tomography
SANTE =: Stimulation of the Anterior Nucleus of the Thalamus for Epilepsy study
SNr =: substantia nigra pars reticulata
STN =: subthalamic nucleus
SUDEP =: sudden unexpected death in epilepsy
VNS =: vagal nerve stimulation
VTA =: volume of tissue activated
