# Supplementary material for: Identifying the neural network for neuromodulation in epilepsy through connectomics and graphs
Source: Brain Commun. 2022 Apr 6;4(3):fcac092. doi: 10.1093/braincomms/fcac092 (PMC9123846; doi:10.1093/braincomms/fcac092)

**Supplementary figure 1. Identification of the normative epilepsy DBS network.** Functional connectivity maps of ANT (red), CMT (green), hippocampus (blue), and areas of overlap between all three binarized t-maps (yellow) identify the epilepsy DBS network. The overlapping regions between the three binarized maps (ANT, CMT, and HC) were used to outline the common network implicated in seizure reduction following DBS.


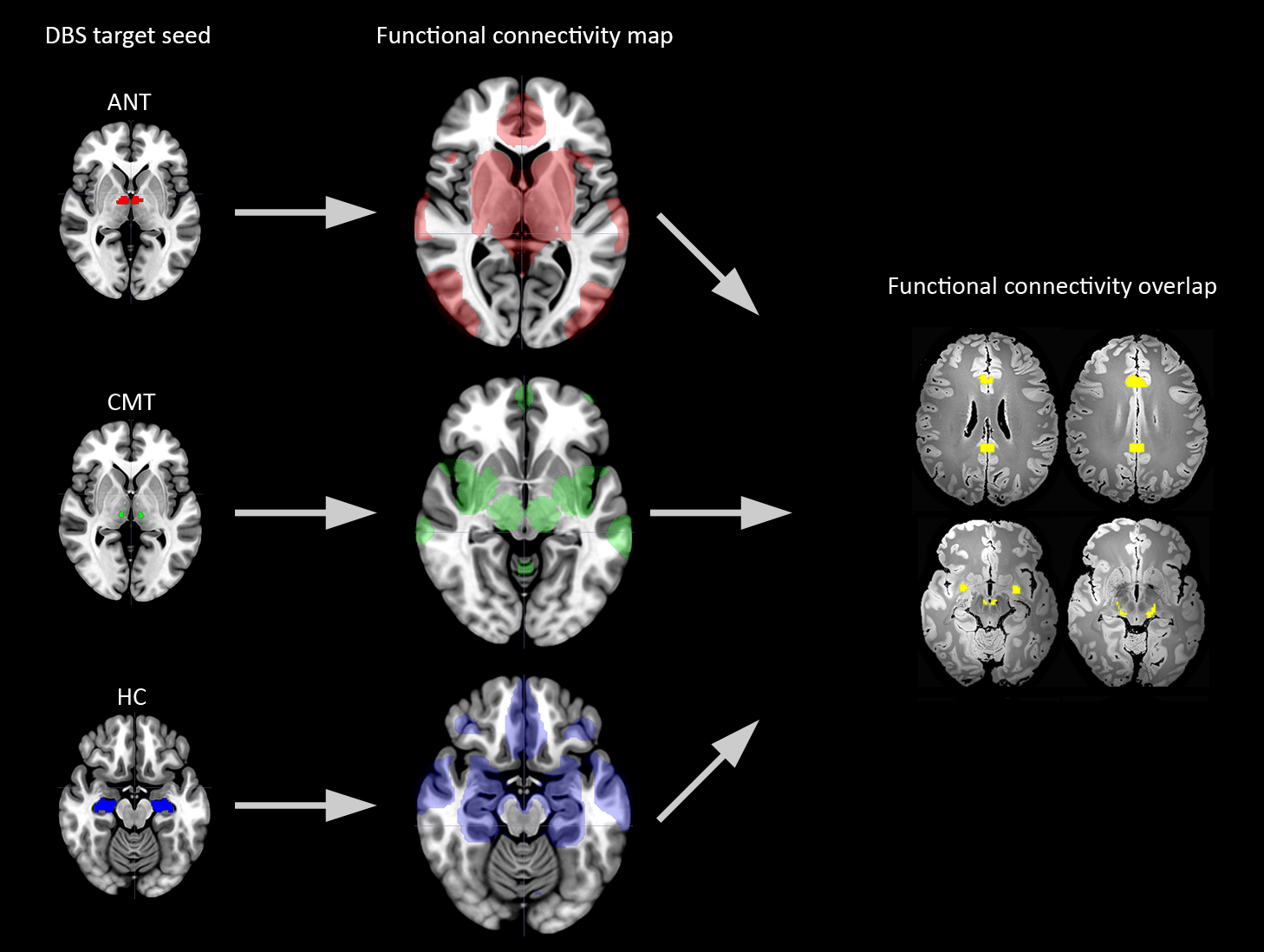

Supplement: fcac092_Supplementary_Data [file fcac092_supplementary_data.zip › Supplementary figure 1.docx]
