# Supplementary material for: Identifying the neural network for neuromodulation in epilepsy through connectomics and graphs
Source: Brain Commun. 2022 Apr 6;4(3):fcac092. doi: 10.1093/braincomms/fcac092 (PMC9123846; doi:10.1093/braincomms/fcac092)
Supplement: fcac092_Supplementary_Data [file fcac092_supplementary_data.zip › Supplementary figure 2.docx]

**Supplementary figure 2.** A simple Pearson correlation matrix of normative functional connectivity between seeds (ANT, CMT, HC, areas of common functional connectivity, less studied and hypothetical DBS targets) without clustering.


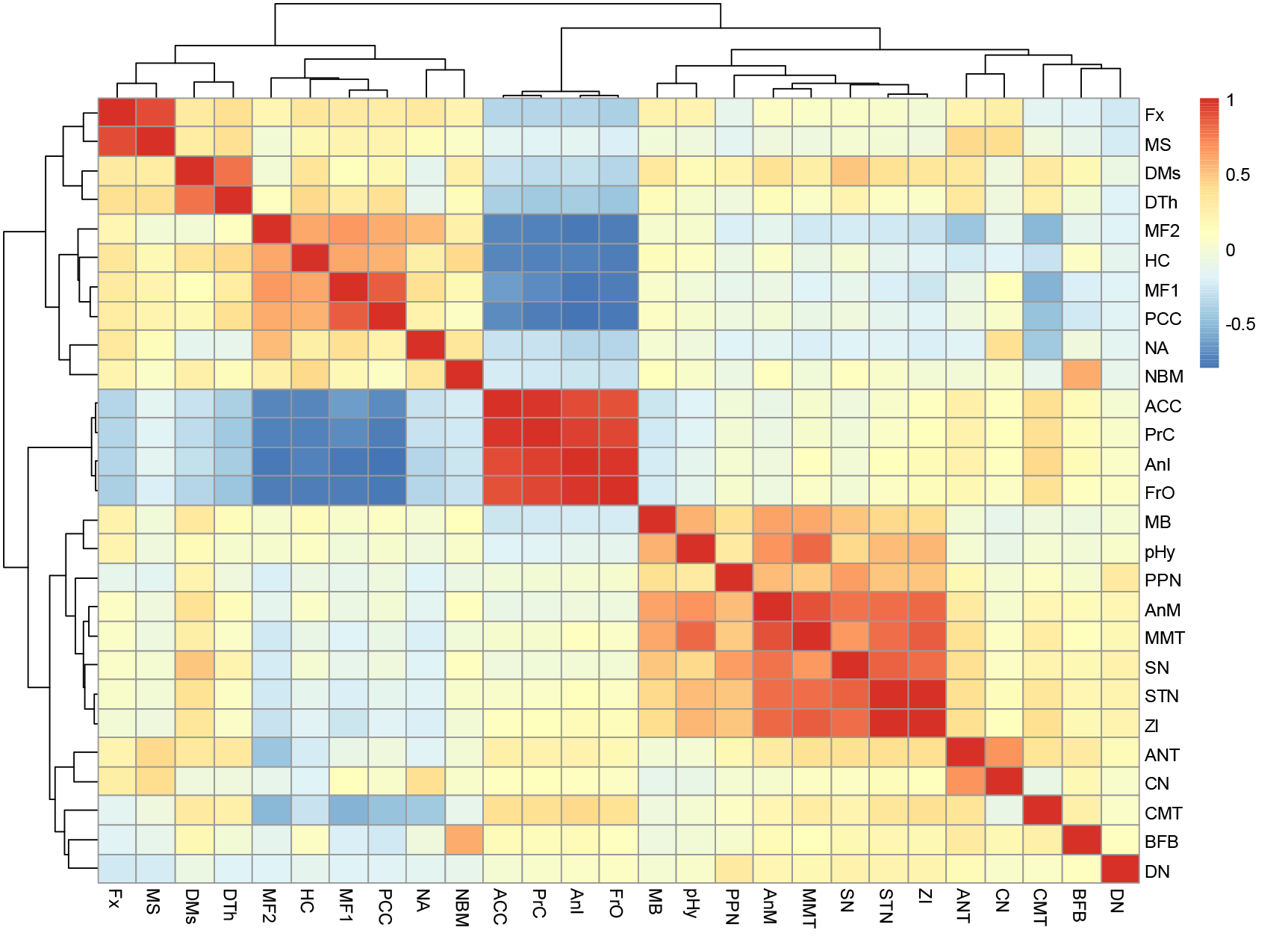


Abbreviations: anterior thalamic nucleus (ANT), centromedian thalamic nucleus (CMT), hippocampus (HC), subthalamic nucleus (STN), substantia nigra pars reticulata (SN), zona incerta (ZI), posterior hypothalamus (PHy), fornix (Fx), nucleus accumbens (NA), head of caudate nucleus (CN), dentate nucleus (DN), mammillothalamic tract (MMT), mammillary body (MB), nucleus basalis of Meynert (NBM), pedunculopontine nucleus (PPN), medial septum (MS), anterior cingulate cortex (ACC), paracingulate cortex (PrC), medial frontal region (MF1 and MF2), posterior cingulate cortex (PCC), anterior insula (AnI), frontal operculum (FrO), basal forebrain (BFB), dorsal thalamus (DTh), dorsal (DMs) and ventral mesencephalon (AnM).
