# Supplementary material for: Identifying the neural network for neuromodulation in epilepsy through connectomics and graphs
Source: Brain Commun. 2022 Apr 6;4(3):fcac092. doi: 10.1093/braincomms/fcac092 (PMC9123846; doi:10.1093/braincomms/fcac092)
Supplement: fcac092_Supplementary_Data [file fcac092_supplementary_data.zip › Supplementary figure 3.docx]

**Supplementary figure 3.** Graph of the brain regions involved in DBS stimulation for epilepsy (ANT, CMT, HC, and areas of their common functional connectivity; other DBS targets included). Thickness of edges represents the strength of correlations between the nodes (minimum correlations presented >0.2).
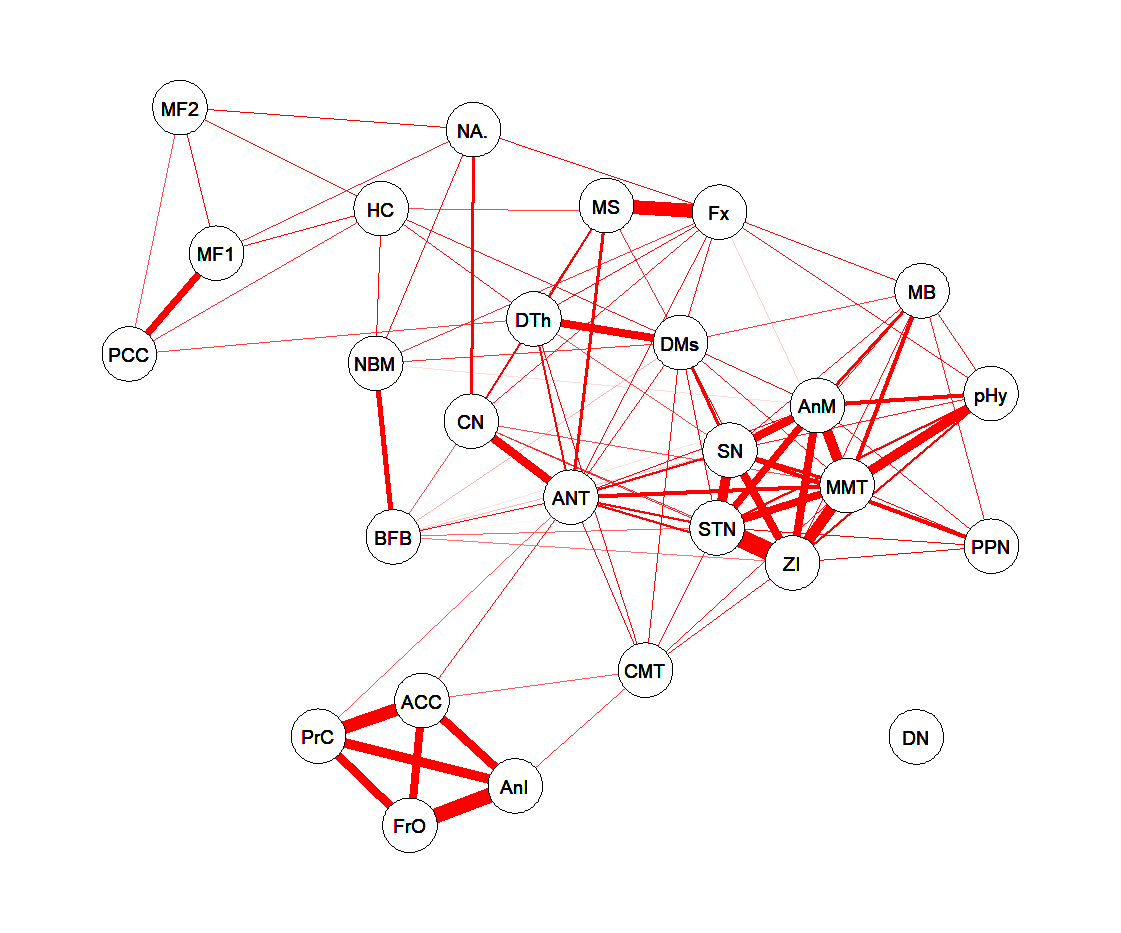


Abbreviations: anterior thalamic nucleus (ANT), centromedian thalamic nucleus (CMT), hippocampus (HC), subthalamic nucleus (STN), substantia nigra pars reticulata (SN), zona incerta (ZI), posterior hypothalamus (PHy), fornix (Fx), nucleus accumbens (NA), head of caudate nucleus (CN), dentate nucleus (DN), mammillothalamic tract (MMT), mammillary body (MB), nucleus basalis of Meynert (NBM), pedunculopontine nucleus (PPN), medial septum (MS), anterior cingulate cortex (ACC), paracingulate cortex (PrC), medial frontal region (MF1 and MF2), posterior cingulate cortex (PCC), anterior insula (AnI), frontal operculum (FrO), basal forebrain (BFB), dorsal thalamus (DTh), dorsal (DMs) and ventral mesencephalon (AnM).
