# Supplementary material for: Identifying the neural network for neuromodulation in epilepsy through connectomics and graphs
Source: Brain Commun. 2022 Apr 6;4(3):fcac092. doi: 10.1093/braincomms/fcac092 (PMC9123846; doi:10.1093/braincomms/fcac092)
Supplement: fcac092_Supplementary_Data [file fcac092_supplementary_data.zip › Supplementary material 1 updated.docx]

**Supplementary material 1.**

**List of epilepsy DBS studies used for calculation of mean seizure reduction:**

**ANT** – Salanova et al. 2021^1^, Schaper et al. 2021^2^, Järvenpää et al. 2020^3^, Kaufmann et al. 2020^4^, Guo et al. 2020^5^, Tassigny et al. 2020^6^, Koeppen et al. 2019^7^, Park et al. 2019^8^, Sitnikov 2018^9^, Herrman et al. 2018^10^, Kim et al. 2017^11^, Krishna et al. 2016^12^, Salanova et al. 2015^13^, Piacentino et al. 2015^14^, Lee et al. 2012^15^, Oh et al. 2011^16^, Fisher et al. 2010^17^, Osorio et al. 2007^18^, Lim et al. 2007^19^, Andrade et al. 2006^20^, Lee et al. 2006^21^, Kerrigan et al. 2004^22^, Hodaie et al. 2002^23^.

**CMT** - Alcala et al. 2021^24^, Cukiert et al. 2020^25^, Kim et al. 2017^26^, Son et al. 2016^27^, Valentin et al. 2013^28^, Cukiert et al. 2009^29^, Velasco et al. 2006^30^, Velasco et al. 2000^31^.

**HC** - Wang et al. 2021^32^, Vasquez et al. 2020^33^, Cukiert et al. 2020^25^, Cukiert et al. 2017^34^, Lim et al. 2016^35^, Jin et al. 2016^36^, Cukiert et al. 2014^37^, Bondallaz et al. 2013^38^, Vonck et al. 2013^39^, Boon et al. 2007^40^, Velasco et al. 2007^41^, Tellez‐Zenteno et al. 2006^42^, Vonck et al. 2002^43^.

**Hypothetical targets** – Gonzalez et al 2021^44^, Hristova et al 2021^45^, Takeuchi et al. 2021^46^, Magloire et al. 2021^47^, Want et al. 2021^48^, Wicker et al. 2019^49^, Balak et al. 2018^50^, Jaseja et al. 2013^51^.

**Less used DBS targets**^52,53^

**Atlases used for the creation of epilepsy DBS target seeds:**

ANT – Atlas of the Basal Ganglia and Thalamus (ABGT)^54^, HC head - Melbourne Subcortex Atlas (Tian 2020)^55^, CMT – Thomas Atlas^56^, cZI, STN – Distal^57^, SN, Fx, NBM, MMT, pHyp – Atlas of the Human Hypothalamus (Neudorfer & Germann 2020)^58^, PPN – PPN Atlas (Alho 2017)^59^, MB – Human Motor Thalamus (Ilinsky 2017)^60^, NA - MNI PD25 subcortical (Xiao 2017)^61^, DN^62^, CN and MS seeds were manually created on the MNI152 standard-space T1-weighted average structural template image using FSLeyes (version 5.0.10).

**Bibliography**

1. Salanova V, Sperling MR, Gross RE, et al. The SANTÉ study at 10 years of follow‐up: Effectiveness, safety, and sudden unexpected death in epilepsy. *Epilepsia*. Published online 2021. doi:10.1111/epi.16895

2. Schaper FLWVJ, Plantinga BR, Colon AJ, et al. Deep brain stimulation in epilepsy: A role for modulation of the mammillothalamic tract in seizure control? *Neurosurgery*. 2020;87(3):602-610. doi:10.1093/neuros/nyaa141

3. Järvenpää S, Lehtimäki K, Rainesalo S, Möttönen T, Peltola J. Improving the effectiveness of ANT DBS therapy for epilepsy with optimal current targeting. *Epilepsia Open*. 2020;5(3):406-417. doi:10.1002/epi4.12407

4. Kaufmann E, Bötzel K, Vollmar C, Mehrkens JH, Noachtar S. What have we learned from 8 years of deep brain stimulation of the anterior thalamic nucleus? Experiences and insights of a single center. *J Neurosurg*. 2021;135(2):619-628. doi:10.3171/2020.6.JNS20695

5. Guo W, Koo BB, Kim JH, et al. Defining the optimal target for anterior thalamic deep brain stimulation in patients with drug-refractory epilepsy. *J Neurosurg*. 2021;134(4):1054-1063. doi:10.3171/2020.2.JNS193226

6. Tassigny D, Soler-Rico M, Delavallée M, Santos SF, El Tahry R, Raftopoulos C. Anterior thalamic nucleus deep brain stimulation for refractory epilepsy: Preliminary results in our first 5 patients. *Neurochirurgie*. 2020;66(4):252-257. doi:10.1016/j.neuchi.2020.03.001

7. Koeppen JA, Nahravani F, Kramer M, et al. Electrical Stimulation of the Anterior Thalamus for Epilepsy: Clinical Outcome and Analysis of Efficient Target. *Neuromodulation*. 2019;22(4):465-471. doi:10.1111/ner.12865

8. Park HR, Choi SJ, Joo EY, et al. The Role of Anterior Thalamic Deep Brain Stimulation as an Alternative Therapy in Patients with Previously Failed Vagus Nerve Stimulation for Refractory Epilepsy. *Stereotact Funct Neurosurg*. 2019;97(3):176-182. doi:10.1159/000502344

9. Sitnikov AR, Grigoryan YA, Mishnyakova LP. Bilateral stereotactic lesions and chronic stimulation of the anterior thalamic nuclei for treatment of pharmacoresistant epilepsy. *Surg Neurol Int*. 2018;9(1). doi:10.4103/sni.sni_25_18

10. Herrman H, Egge A, Konglund AE, Ramm‐Pettersen J, Dietrichs E, Taubøll E. Anterior thalamic deep brain stimulation in refractory epilepsy: A randomized, double‐blinded study. *Acta Neurol Scand*. 2018;139(3):ane.13047. doi:10.1111/ane.13047

11. Kim SH, Lim SC, Kim J, Son BC, Lee KJ, Shon YM. Long-term follow-up of anterior thalamic deep brain stimulation in epilepsy: A 11-year, single center experience. *Seizure*. 2017;52:154-161. doi:10.1016/j.seizure.2017.10.009

12. Krishna V, King NKK, Sammartino F, et al. Anterior nucleus deep brain stimulation for refractory epilepsy: Insights into patterns of seizure control and efficacious target. *Neurosurgery*. 2016;78(6):802-811. doi:10.1227/NEU.0000000000001197

13. Salanova V, Witt T, Worth R, et al. Long-term efficacy and safety of thalamic stimulation for drug-resistant partial epilepsy. *Neurology*. 2015;84(10):1017-1025. doi:10.1212/WNL.0000000000001334

14. Piacentino M, Durisotti C, Garofalo PG, et al. Anterior thalamic nucleus deep brain Stimulation (DBS) for drug-resistant complex partial seizures (CPS) with or without generalization: long-term evaluation and predictive outcome. *Acta Neurochir (Wien)*. 2015;157(9):1525-1532. doi:10.1007/s00701-015-2498-1

15. Lee KJ, Shon YM, Cho CB. Long-term outcome of anterior thalamic nucleus stimulation for intractable epilepsy. *Stereotact Funct Neurosurg*. 2012;90(6):379-385. doi:10.1159/000339991

16. Oh YS, Kim HJ, Lee KJ, Kim YI, Lim SC, Shon YM. Cognitive improvement after long-term electrical stimulation of bilateral anterior thalamic nucleus in refractory epilepsy patients. *Seizure*. 2012;21(3):183-187. doi:10.1016/j.seizure.2011.12.003

17. Fisher R, Salanova V, Witt T, et al. Electrical stimulation of the anterior nucleus of thalamus for treatment of refractory epilepsy. *Epilepsia*. 2010;51(5):899-908. doi:10.1111/j.1528-1167.2010.02536.x

18. Osorio I, Overman J, Giftakis J, Wilkinson SB. High Frequency Thalamic Stimulation for Inoperable Mesial Temporal Epilepsy. *Epilepsia*. 2007;48(8):1561-1571. doi:10.1111/j.1528-1167.2007.01044.x

19. Lim SN, Lee ST, Tsai YT, et al. Electrical Stimulation of the Anterior Nucleus of the Thalamus for Intractable Epilepsy: A Long-term Follow-up Study. *Epilepsia*. 2007;48(2):342-347. doi:10.1111/j.1528-1167.2006.00898.x

20. Andrade DM, Zumsteg D, Hamani C, et al. Long-term follow-up of patients with thalamic deep brain stimulation for epilepsy. *Neurology*. 2006;66(10):1571-1573. doi:10.1212/01.wnl.0000206364.19772.39

21. Lee KJ, Jang KS, Shon YM. Chronic deep brain stimulation of subthalamic and anterior thalamic nuclei for controlling refractory partial epilepsy. *Acta Neurochir Suppl*. 2006;(99):87-91. doi:10.1007/978-3-211-35205-2_17

22. Kerrigan JF, Litt B, Fisher RS, et al. Electrical Stimulation of the Anterior Nucleus of the Thalamus for the Treatment of Intractable Epilepsy. *Epilepsia*. 2004;45(4):346-354. doi:10.1111/j.0013-9580.2004.01304.x

23. Hodaie M, Wennberg RA, Dostrovsky JO, Lozano AM. Chronic anterior thalamus stimulation for intractable epilepsy. *Epilepsia*. 2002;43(6):603-608. doi:10.1046/j.1528-1157.2002.26001.x

24. Alcala-Zermeno JL, Gregg NM, Wirrell EC, et al. Centromedian thalamic nucleus with or without anterior thalamic nucleus deep brain stimulation for epilepsy in children and adults: A retrospective case series. *Seizure*. 2021;84:101-107. doi:10.1016/j.seizure.2020.11.012

25. Cukiert A, Cukiert CM, Burattini JA, Mariani PP. Seizure outcome during bilateral, continuous, thalamic centromedian nuclei deep brain stimulation in patients with generalized epilepsy: a prospective, open-label study. *Seizure*. 2020;81(September):304-309. doi:10.1016/j.seizure.2020.08.028

26. Kim SH, Lim SC, Yang DW, et al. Thalamo–cortical network underlying deep brain stimulation of centromedian thalamic nuclei in intractable epilepsy: A multimodal imaging analysis. *Neuropsychiatr Dis Treat*. 2017;13:2607-2619. doi:10.2147/NDT.S148617

27. Son BC, Shon YM, Choi JG, et al. Clinical Outcome of Patients with Deep Brain Stimulation of the Centromedian Thalamic Nucleus for Refractory Epilepsy and Location of the Active Contacts. *Stereotact Funct Neurosurg*. 2016;94(3):187-197. doi:10.1159/000446611

28. Valentín A, García Navarrete E, Chelvarajah R, et al. Deep brain stimulation of the centromedian thalamic nucleus for the treatment of generalized and frontal epilepsies. *Epilepsia*. 2013;54(10):1823-1833. doi:10.1111/epi.12352

29. Cukiert A, Burattini JA, Cukiert CM, et al. Centro-median stimulation yields additional seizure frequency and attention improvement in patients previously submitted to callosotomy. *Seizure*. 2009;18(8):588-592. doi:10.1016/j.seizure.2009.06.002

30. Velasco AL, Velasco F, Jiménez F, et al. Neuromodulation of the centromedian thalamic nuclei in the treatment of generalized seizures and the improvement of the quality of life in patients with Lennox-Gastaut syndrome. *Epilepsia*. 2006;47(7):1203-1212. doi:10.1111/j.1528-1167.2006.00593.x

31. Velasco F, Velasco M, Jiménez F, et al. Predictors in the treatment of difficult-to-control seizures by electrical stimulation of the centromedian thalamic nucleus. *Neurosurgery*. 2000;47(2):295-305. doi:10.1097/00006123-200008000-00007

32. Wang S, Zhao M, Li T, et al. Long-term efficacy and cognitive effects of bilateral hippocampal deep brain stimulation in patients with drug-resistant temporal lobe epilepsy. *Neurol Sci*. 2021;42(1):225-233. doi:10.1007/s10072-020-04554-8

33. Vázquez-Barrón D, Cuéllar-Herrera M, Velasco F, Velasco AL. Electrical Stimulation of Subiculum for the Treatment of Refractory Mesial Temporal Lobe Epilepsy with Hippocampal Sclerosis: A 2-Year Follow-Up Study. *Stereotact Funct Neurosurg*. 2021;99(1):40-47. doi:10.1159/000510295

34. Cukiert A, Cukiert CM, Burattini JA, et al. Seizure outcome after hippocampal deep brain stimulation in patients with refractory temporal lobe epilepsy: A prospective, controlled, randomized, double-blind study. *Epilepsia*. 2017;58(10):1728-1733. doi:10.1111/epi.13860

35. Lim SN, Lee CY, Lee ST, et al. Low and High Frequency Hippocampal Stimulation for Drug-Resistant Mesial Temporal Lobe Epilepsy. *Neuromodulation*. 2016;19(4):365-372. doi:10.1111/ner.12435

36. Jin H, Li W, Dong C, et al. Hippocampal deep brain stimulation in nonlesional refractory mesial temporal lobe epilepsy. *Seizure*. 2016;37(348):1-7. doi:10.1016/j.seizure.2016.01.018

37. Cukiert A, Cukiert CM, Burattini JA, Lima AM. Seizure outcome after hippocampal deep brain stimulation in a prospective cohort of patients with refractory temporal lobe epilepsy. *Seizure*. 2014;23(1):6-9. doi:10.1016/j.seizure.2013.08.005

38. Bondallaz P, Boëx C, Rossetti AO, et al. Electrode location and clinical outcome in hippocampal electrical stimulation for mesial temporal lobe epilepsy. *Seizure*. 2013;22(5):390-395. doi:10.1016/j.seizure.2013.02.007

39. Vonck K, Sprengers M, Carrette E, et al. A decade of experience with deep brain stimulation for patients with refractory medial temporal lobe epilepsy. *Int J Neural Syst*. 2013;23(1). doi:10.1142/S0129065712500347

40. Boon P, Vonck K, De Herdt V, et al. Deep brain stimulation in patients with refractory temporal lobe epilepsy. *Epilepsia*. 2007;48(8):1551-1560. doi:10.1111/j.1528-1167.2007.01005.x

41. Velasco AL, Velasco F, Velasco M, Trejo D, Castro G, Carrillo-Ruiz JD. Electrical stimulation of the hippocampal epileptic foci for seizure control: A double-blind, long-term follow-up study. *Epilepsia*. 2007;48(10):1895-1903. doi:10.1111/j.1528-1167.2007.01181.x

42. Tellez-Zenteno JF, McLachlan RS, Parrent A, et al. Hippocampal electrical stimulation in mesial temporal lobe epilepsy. *Neurology*. 2006;66(10):1490-1494. doi:10.1212/01.wnl.0000209300.49308.8f

43. Vonck K, Boon P, Achten E, De Reuck J, Caemaert J. Long-term amygdalohippocampal stimulation for refractory temporal lobe epilepsy. *Ann Neurol*. 2002;52(5):556-565. doi:10.1002/ana.10323

44. González HFJ, Narasimhan S, Johnson GW, et al. Role of the Nucleus Basalis as a Key Network Node in Temporal Lobe Epilepsy. *Neurology*. 2021;96(9):e1334-e1346. doi:10.1212/WNL.0000000000011523

45. Hristova K, Martinez-Gonzalez C, Watson TC, et al. Medial septal GABAergic neurons reduce seizure duration upon optogenetic closed-loop stimulation. *Brain*. 2021;144(5):1576-1589. doi:10.1093/BRAIN/AWAB042

46. Takeuchi Y, Harangozó M, Pedraza L, et al. Closed-loop stimulation of the medial septum terminates epileptic seizures. *Brain*. 2021;144(3):885-908. doi:10.1093/brain/awaa450

47. Magloire V, Lignani G. DBS for refractory epilepsy: is closed-loop stimulation of the medial septum the way forward? *Brain*. 2021;144(3):702-705. doi:10.1093/BRAIN/AWAB051

48. Wang Y, Shen Y, Cai X, et al. Deep brain stimulation in the medial septum attenuates temporal lobe epilepsy via entrainment of hippocampal theta rhythm. *CNS Neurosci Ther*. 2021;27(5):577-586. doi:10.1111/cns.13617

49. Wicker E, Beck VC, Kulick-Soper C, et al. Descending projections from the substantia nigra pars reticulata differentially control seizures. *Proc Natl Acad Sci U S A*. 2019;116(52):27084-27094. doi:10.1073/pnas.1908176117

50. Balak N, Balkuv E, Karadag A, et al. Mammillothalamic and Mammillotegmental Tracts as New Targets for Dementia and Epilepsy Treatment. *World Neurosurg*. 2018;110:133-144. doi:10.1016/j.wneu.2017.10.168

51. H J. Pedunculopontine nucleus stimulation in intractable epilepsy: simulation of nature’s antiepileptic role and mechanism. *Epilepsy Behav*. 2013;27(3):507. doi:10.1016/J.YEBEH.2013.03.004

52. Zangiabadi N, Ladino LDi, Sina F, Orozco-Hernández JP, Carter A, Téllez-Zenteno JF. Deep brain stimulation and drug-resistant epilepsy: A review of the literature. *Front Neurol*. 2019;10(JUN):1-18. doi:10.3389/fneur.2019.00601

53. Li MCH, Cook MJ. Deep brain stimulation for drug-resistant epilepsy. *Epilepsia*. 2018;59(2):273-290. doi:10.1111/EPI.13964

54. He X, Chaitanya G, Asma B, et al. Disrupted basal ganglia–thalamocortical loops in focal to bilateral tonic-clonic seizures. *Brain*. 2020;143(1):175-190. doi:10.1093/BRAIN/AWZ361

55. Tian Y, Margulies DS, Breakspear M, Zalesky A. Topographic organization of the human subcortex unveiled with functional connectivity gradients. *Nat Neurosci 2020 2311*. 2020;23(11):1421-1432. doi:10.1038/s41593-020-00711-6

56. Su JH, Thomas FT, Kasoff WS, et al. Thalamus Optimized Multi Atlas Segmentation (THOMAS): fast, fully automated segmentation of thalamic nuclei from structural MRI. *Neuroimage*. 2019;194:272-282. doi:10.1016/J.NEUROIMAGE.2019.03.021

57. Ewert S, Plettig P, Li N, et al. Toward defining deep brain stimulation targets in MNI space: A subcortical atlas based on multimodal MRI, histology and structural connectivity. *Neuroimage*. 2018;170:271-282. doi:10.1016/J.NEUROIMAGE.2017.05.015

58. Neudorfer C, Germann J, Elias GJB, Gramer R, Boutet A, Lozano AM. A high-resolution in vivo magnetic resonance imaging atlas of the human hypothalamic region. *Sci Data 2020 71*. 2020;7(1):1-14. doi:10.1038/s41597-020-00644-6

59. Alho ATDL, Hamani C, Alho EJL, et al. Magnetic resonance diffusion tensor imaging for the pedunculopontine nucleus: proof of concept and histological correlation. *Brain Struct Funct 2017 2226*. 2017;222(6):2547-2558. doi:10.1007/S00429-016-1356-0

60. Ilinsky I, Horn A, Paul-Gilloteaux P, Gressens P, Verney C, Kultas-Ilinsky K. Human Motor Thalamus Reconstructed in 3D from Continuous Sagittal Sections with Identified Subcortical Afferent Territories. *eNeuro*. 2018;5(3):60-78. doi:10.1523/ENEURO.0060-18.2018

61. Xiao Y, Fonov V, Chakravarty MM, et al. A dataset of multi-contrast population-averaged brain MRI atlases of a Parkinson׳s disease cohort. *Data Br*. 2017;12:370-379. doi:10.1016/J.DIB.2017.04.013

62. Diedrichsen J, Maderwald S, Küper M, et al. Imaging the deep cerebellar nuclei: A probabilistic atlas and normalization procedure. *Neuroimage*. 2011;54(3):1786-1794. doi:10.1016/j.neuroimage.2010.10.035
