# Supplementary material for: Identifying the neural network for neuromodulation in epilepsy through connectomics and graphs
Source: Brain Commun. 2022 Apr 6;4(3):fcac092. doi: 10.1093/braincomms/fcac092 (PMC9123846; doi:10.1093/braincomms/fcac092)
Supplement: fcac092_Supplementary_Data [file fcac092_supplementary_data.zip › Supplementary table 1.docx]

**Supplementary table 1.** **Areas of functional connectivity overlap between binarized t-maps of ANT, CMT, HC and other DBS targets.** Functional connectivity of less used and hypothetical epilepsy DBS targets was assessed and compared with the regions included in the epilepsy DBS network (*hypothetical targets).

| Areas of FC overlap between ANT, CMT and HC | Other targets with FC overlap in the same region |
| --- | --- |
| Cortical structures | |
| Anterior cingulate and paracingulate cortex | ZI, STN, SN, HCN, MMT |
| Medial frontal region | NA, Fx, HCN, MS*, NBM* |
| Posterior cingulate | ‐ |
| Frontal operculum | ‐ |
| Sensorimotor cortex (right) | SN, HCN, MMT |
| Bilateral insulae | ZI, HCN, MMT |
| Subcortical structures | |
| Medial thalamus | NA, Fx, ZI, STN, SN, HCN, DN MMT, PPN*, MS*, NBM* |
| Dorsal thalamus | Fx, ZI STN, SN, MMT, MS*, MB* |
| Dorsal mesencephalon | Fx, ZI, STN, SN, MMT, PPN*, NBM*, MS*, MB* |
| Anterior mesencephalon | Fx, ZI, STN, SN, HCN, DN, MMT, PH, PPN*, MB*, NBM* |
| Basal forebrain | ZI, STN, SN, HCN, MMT, NBM* |

Abbreviations: anterior thalamic nucleus (ANT), centromedian thalamic nucleus (CMT), hippocampus (HC), subthalamic nucleus (STN), substantia nigra (SN), caudal zona incerta (cZI), posterior hypothalamus (PH), fornix (Fx), nucleus accumbens (NA), head of caudate nucleus (HCN), dentate nucleus (DN), mammillothalamic tract (MMT), mammillary body (MB), nucleus basalis of Meynert (NBM), pedunculopontine nucleus (PPN), medial septum (MS).
